# Clarifying the distinction between case series and cohort studies in systematic reviews of comparative studies: potential impact on body of evidence and workload

**DOI:** 10.1186/s12874-017-0391-8

**Published:** 2017-07-17

**Authors:** Tim Mathes, Dawid Pieper

**Affiliations:** Institute for Research in Operative Medicine, Chair of Surgical Research, Faculty of Health, School of Medicine, Witten/Herdecke University, Ostmerheimer Str. 200, 51109 Cologne, Germany

## Abstract

Distinguishing cohort studies from case series is difficult.

We propose a conceptualization of cohort studies in systematic reviews of comparative studies. The main aim of this conceptualization is to clarify the distinction between cohort studies and case series. We discuss the potential impact of the proposed conceptualization on the body of evidence and workload.

All studies with exposure-based sampling gather multiple exposures (with at least two different exposures or levels of exposure) and enable calculation of relative risks that should be considered cohort studies in systematic reviews, including non-randomized studies. The term “enables/can” means that a predefined analytic comparison is not a prerequisite (i.e., the absolute risks per group and/or a risk ratio are provided). Instead, all studies for which sufficient data are available for reanalysis to compare different exposures (e.g., sufficient data in the publication) are classified as cohort studies.

There are possibly large numbers of studies without a comparison for the exposure of interest but that do provide the necessary data to calculate effect measures for a comparison. Consequently, more studies could be included in a systematic review. Therefore, on the one hand, the outlined approach can increase the confidence in effect estimates and the strengths of conclusions. On the other hand, the workload would increase (e.g., additional data extraction and risk of bias assessment, as well as reanalyses).

## Background

Systematic reviews that include non-randomized studies often consider different observational study designs [1]. However, the distinction between different non-randomized study designs is difficult. One key design feature to classify observational study designs is to distinguish comparative from non-comparative studies [2, 3]. The lack of a comparison group is of particular importance for distinguishing cohort studies from case series because in many definitions, they share a main design feature of having a follow-up period examining the exposed individuals over time [2, 3]. The only difference between cohort studies and case series in many definitions is that cohort studies compare different groups (i.e., examine the association between exposure and outcome), while case series are uncontrolled [3–5]. Table 1 shows an example definition [3]. The problem with this definition is that vague terms, such as comparison and examination of association, might be interpreted as an analytic comparison of at least two exposures (i.e., interventions, risk factors or prognostic factors).

**Table 1:** Example definitions of cohort studies and case series [2]

*Cohort study*
A study in which a defined group of people (the cohort) is followed over time, to *examine* associations between different interventions received and subsequent outcomes.
*Case series*
Observations are made on a series of individuals, usually all receiving the same intervention, before and after an intervention but with no control group.

**Fig. 1:**
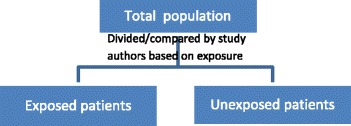
Cohort study (vague definition)

For example, imagine a study of 20 consecutive patients with a certain disease that can be treated in two different ways. A study that divides the 20 patients into two groups according to the treatment received and compares the outcomes of these groups (e.g., provides aggregated absolute risks per group or a risk ratio) would be probably classified as a cohort study (the example used in the following sections is denoted “study 1”). A sample of this study type is illustrated in Fig. 1 and Table 2.

**Table 2:** Possible presentation of a study with a preexisting exposure based comparison (cohort study not requiring a reanalysis)

Group 1	Group 2	Effect
Absolute risk for outcome (e.g. mortality rate) in all exposed (e.g. treated) patients	Absolute risk for outcome (e.g. mortality rate) in all unexposed (e.g. not treated) patients	Risk ratio with 95% confidence interval (e.g. relative risk of mortality for treated vs. untreated)

**Fig. 2:**
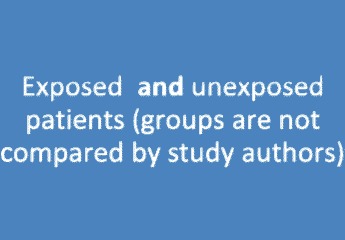
Case series (vague definition)

In contrast, a publication that describes the interventions received and outcomes for each patient/case separately would probably be classified as a case series (the example in the following sections is denoted “study 2”). An example of this study type is illustrated in Fig. 2 and Table 3. In the medical literature, the data on exposure and outcomes are usually provided in either running text or spreadsheet formats [6–21]. A good example is the study by Wong et al. [10]. In this study, information on placental invasion (exposure) and blood loss (outcome) is separately provided for 40 pregnant women in a table. The study by Cheng et al. is an example of a study providing information in the running text (i.e., anticoagulation management [exposure] and recovery [outcome] for paediatric stroke) [6].

**Table 3:** Possible presentation of study without a preexisting exposure based comparison (cohort study requiring a reanalysis)

Patient	Exposure	Outcome
Patient 1	Yes (e.g. treated)	Yes (e.g. died)
Patient 2	No (e.g. not treated)	No (e.g. alive)
.	.	.
.	.	.
.	.	.
Patient 20	Yes (e.g. treated)	Yes (e.g. alive)

These examples illustrate that distinguishing between cohort studies and case series is difficult. Vague definitions are probably the reason for the common confusion between study designs. A recent study found that approximately 72% of cohort studies are mislabelled as case series [22]. Many systematic reviews of non-randomized studies included cohort studies but excluded case series (see examples in [23–28]). Therefore, the unclear distinction between case series and cohort studies can result in inconsistent study selection and unjustified exclusions from a systematic review. The risk of misclassification is particularly high because study authors also often mislabel their study or studies are not classified by their authors at all (see examples in [6–21]).

## Objective

We propose a conceptualization of cohort studies in systematic reviews of comparative studies. The main objective of this conceptualization is to clarify the distinction between cohort studies and case series in systematic reviews, including non-randomized comparative studies. We discuss the potential impact of the proposed conceptualization on the body of evidence and workload.

## Clarifying the distinction between case series and cohort studies (the solution)

In the following report, we propose a conceptualization for cohort studies and case series (e.g., sampling) for systematic reviews, including comparative non-randomized studies. Our proposal is based on a recent conceptualization of cohort studies and case series by Dekkers et al. [29]. The main feature of this conceptualization is that it is exclusively based on inherent design features and is not affected by the analysis.

### Cohort studies of one exposure/one group

Dekkers et al. [29] defined cohort studies with one exposure as studies with exposure-based sampling that enable calculating absolute effects measures for a risk of outcome. This definition means that “the absence of a control group in an exposure-based study does not define a case series” [29]. The definition of cohort studies according to Dekkers et al. [29] is summarized in Table 4.

**Table 4:** Summary of the distinction proposed by Dekkers et al. [28]

*Cohort study*: Patients are sampled on the basis of exposure. The occurrence of outcomes is assessed during a specified follow-up period.
*Case series:* Patients with a particular disease or disease-related outcome are sampled. Case series exist in 2 types:
*1. Sampling* is based on a specific outcome and presence of a specific exposure.
*2. Selection* is based only on a specific outcome, and data are collected on previous exposures. Cases are reported regardless of whether they have specific exposures. This type of case series can be seen as the case group from a case–control study .

### Cohort studies of multiple exposures/more than one group

**Fig. 3:**
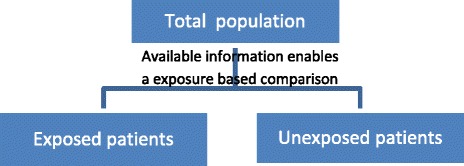
Cohort study (deduced from Dekkers et al. [28])

This idea can be easily extended to studies with more than one exposure. In this case, all studies with exposure-based sampling gathering multiple exposures (i.e., at least two different exposures, manifestations of exposures or levels of exposures) can be considered as (comparative) cohort studies (Fig. 3). The sampling is based on exposure, and there are different groups. Consequently, relative risks can be calculated [29]. The term “enables/can” implies that a predefined analytic comparison is not a prerequisite but that all studies with sufficient data to enable a reanalysis (e.g., in the publication, study reports, and supplementary material) would be classified as cohort studies.

In short, all studies that enable calculation of a relative risk to quantify a difference in outcomes between different groups should be considered cohort studies.

### Case series

**Fig. 4:**
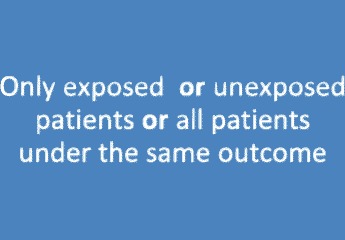
Case series (Deckers et al. [28])

According to Dekkers et al. [29], the sampling of a case series is either based on exposure and outcome (e.g., all patients are treated and have an adverse event) or case series include patients with a certain outcome regardless of exposure (see Fig. 4). Consequently, no absolute risk and also no relative effect measures for an outcome can be calculated in a case series. Note that sampling in a case series does not need to be consecutive. Consecutiveness would increase the quality of the case series, but a non-consecutive series is also a case series [29].

In short, for a case series, there are no absolute risks, and also, no risk ratios can be calculated. Consequently, a case series cannot be comparative. The definition of a case series by Dekkers et al. [29] is summarized in Table 4.

It is noteworthy that the conceptualization also ensures a clear distinction of case series from other study designs that apply outcome-based sampling. Case series, case-control studies (including case-time-control), and self-controlled case-control designs (e.g., case-crossover) all have outcome-based sampling in common [29].

Case series have no control at all because only patients with a certain manifestation of outcomes are sampled (e.g., individuals with a disease or deceased individuals). In contrast, all case-control designs as well as self-controlled case-control designs have a control group. In case-control studies, the control group constitutes individuals with another manifestation of the outcome (e.g., healthy individuals or survivors). This outcome can be considered as two case series (i.e., case group and no case group).

Self-controlled case-control studies are characterized by an intra-individual comparison (each individual is their own control) [30]. Information is also sampled when patients are not exposed. Therefore, case-control designs as well as self-controlled case-control studies enable the calculation of risk ratios. This approach is not possible for a case series.

### Illustrating example

Above, we illustrated that by using a vague definition, the classification of a study design might be influenced by the preparation and analysis of the study data. The proposed conceptualization is exclusively based on the inherent design features (e.g., sampling, exposure). After considering the example studies again using the proposed conceptualization, all studies would be classified as cohort studies because the relative risk can be calculated. This outcome becomes clear looking at Table 2 and Table 3. If the patients in Table 3 are rearranged according the exposure and the data are reanalysed (i.e., calculation of absolute risk per group and relative risks to compare groups), Table 3 can be converted into Table 2 (and also, Fig. 2 can be converted to Fig. 3). In the study by Wong et al. [10], the mean blood loss in the group with placental invasion and in the group without placental invasion can be calculated and compared (e.g., relative risk with 95% confidence limits). In this study, the data on gestational age are also provided in the table. Therefore, it is even possible to adjust the results for gestational age (e.g., using a logistic regression).

## Discussion (the impact)

### Influence on the body of evidence

The proposed conceptualization is exclusively based on inherent study design features; therefore, there is less room for misinterpretation compared to existing conceptualizations because analysis features, presentation of data and labelling of the study are not determined. Thus, the conceptualization ensures consistent study selection for systematic reviews.

The prerequisite of an analytical comparison in the publication can lead to the unjustified exclusion of relevant studies from a systematic review. Study 1 would likely be included, and Study 2 would be excluded from the systematic review. The only differences between Study 1 and Study 2 are the analysis and preparation of data. If the data source (e.g., chart review) and the reanalysis (calculation of effect measures and statistical tests) to compare the intervention and control group in Study 2 are performed exactly with the same approach as the existing analysis in Study 1, there can be no difference in the effect estimates between studies, and the studies are at the same risk of bias. Thus, the inclusion of Study 1 and the exclusion of Study 2 are contradictory to the requirement that systematic reviews identify all available evidence [31].

Considering that more studies would be eligible for inclusion and that the hierarchical paradigm of the levels of evidence is not valid per se, the proposed conceptualization can potentially enrich bodies of evidence and increase confidence in effect estimates.

### Influence on workload

The additional inclusion of all studies that enable calculating relative risk for the comparison of interest might impact the workload of systematic reviews. There might be a considerable number of studies not performing a comparison already but that provide sufficient data for reanalysis. Usually the electronic search strategy for systematic reviews of non-randomized studies is not limited to certain study types because there are no sensitive search filters available yet [32]. Therefore, the search results do not usually include cohort studies as discussed above. However, in many abstracts it would be not directly clear if sufficient data for re-calculations are reported in the full text article (e.g., a table like Table 3). Consequently, many additional potentially relevant full-text studies have to be screened. Additionally, studies often assess various exposures (e.g., different baseline characteristics), and it might thus be difficult to identify relevant exposures. Considering the large amount of wrongly labelled studies, this approach can lead to additional screening effort [22].

As a result, more studies would be included in systematic reviews. All articles that provide potentially relevant data would have to be assessed in detail to decide whether reanalysis is feasible. For these data extractions, a risk of bias assessment would have to be performed. Challenges in the risk of bias assessment would arise because most assessment tools are constructed to assess a predefined control group [33]. For example, items regarding the adequacy of analysis (e.g., adjustment for confounders) cannot be assessed anymore. Effect measures must be calculated (e.g., risks by group and relative risk with a 95% confidence limit), and eventually further analyses (e.g., adjustments for confounders) might be necessary for studies that provide sufficient data. Moreover, advanced biometrical expertise would be necessary to judge the feasibility (i.e., determining the possibility to calculate relative risks and whether there are sufficient data to adjust for confounders) of a re-analysis and to conduct the reanalysis.

### Promising areas of application

In the medical literature, it is likely that more retrospective mislabelled cohort studies (comparison planned after data collection) based on routinely collected data (e.g., chart review, review of radiology databases) than prospectively planned (i.e., comparisons planned before data collection) and wrongly labelled cohort studies can be found. Thus, it can be assumed that the wrongly labelled studies tend to have lower methodological quality than studies that already include a comparison. This aspect should be considered in decisions about including studies that must be reanalysed. In research areas in which randomized controlled trials or large planned prospective and well-conducted cohort studies can be expected (e.g., risk factors for widespread diseases), the approach is less promising for enriching the body of evidence. Consequently, in these areas, the additional effort might not be worthwhile.

Again, the conceptualization is particularly promising in research areas in which evidence is sparse because studies are difficult to conduct or populations are small or the event rates are low. These areas include rare diseases, adverse events/complications, sensitive groups (e.g., children or individuals with cognitive deficiencies) or rarely used interventions (e.g., costly innovations). In these areas, there might be no well-conducted studies at all [34, 35]. Therefore, the proposed conceptualization in this report has great potential to increase confidence in effect estimates.

## Conclusion

We proposed a conceptualization for cohort studies with multiple exposures that ensures a clear distinction from case series. In this conceptualization, all studies that contain sufficient data to conduct a reanalysis and not only studies with a pre-existing analytic comparison are classified as cohort studies and are considered appropriate for inclusion in systematic reviews. To the best of our knowledge, no systematic reviews exist that reanalyse (mislabelled) case series to create cohort studies. The outlined approach is a method that can potentially enrich the body of evidence and subsequently enhance confidence in effect estimates and the strengths of conclusions. However, the enrichment of the body of evidence should be balanced against the additional workload.
